# Genetic analysis and QTL mapping of yield and fruit traits in bitter gourd (*Momordica charantia* L.)

**DOI:** 10.1038/s41598-021-83548-8

**Published:** 2021-02-18

**Authors:** P. Gangadhara Rao, T. K. Behera, Ambika B. Gaikwad, A. D. Munshi, Arpita Srivastava, G. Boopalakrishnan, Vinod

**Affiliations:** 1 Division of Vegetable Science, ICAR-Indian Agricultural Research Institute, New Delhi, 110012 India; 2 ICAR-National Bureau of Plant Genetic Resources, New Delhi, 110012 India; 3 Division of Genetics, ICAR-Indian Agricultural Research Institute, New Delhi, 110012 India

**Keywords:** Genetics, Molecular biology, Plant sciences

## Abstract

Bitter gourd (*Momordica charantia* L.) is an economically important vegetable crop grown in tropical parts of the world. In this study, a high-density linkage map of *M. charantia* was constructed through genotyping-by-sequencing (GBS) technology using F_2:3_ mapping population generated from the cross DBGy-201 × Pusa Do Mausami. About 2013 high-quality SNPs were assigned on a total of 20 linkage groups (LGs) spanning over 2329.2 CM with an average genetic distance of 1.16 CM. QTL analysis was performed for six major yield-contributing traits such as fruit length, fruit diameter, fruit weight, fruit flesh thickness, number of fruits per plant and yield per plant. These six quantitative traits were mapped with 19 QTLs (9 QTLs with LOD > 3) using composite interval mapping (CIM). Among 19 QTLs, 12 QTLs derived from ‘Pusa Do Mausami’ revealed a negative additive effect when its allele increased trait score whereas 7 QTLs derived from ‘DBGy-201’ revealed a positive additive effect when its allele trait score increased. The phenotypic variation (R^2^%) elucidated by these QTLs ranged from 0.09% (fruit flesh thickness) on LG 14 to 32.65% (fruit diameter) on LG 16 and a total of six major QTLs detected. Most QTLs detected in the present study were located relatively very close, maybe due to the high correlation among the traits. This information will serve as a significant basis for marker-assisted selection and molecular breeding in bitter gourd crop improvement.

## Introduction

Bitter gourd (syn. bitter melon; *Momordica charantia* L.; 2n = 22) is an economically important vegetable crop and it is abundantly cultivated in India, China, Malaysia, Africa, and South America^1,2^. Indian bitter gourd has a wide phenotypic variation for growth habit, maturity, fruit shape, size, colour, and surface texture^3^ and sex expression^4^. Fruits along with seeds of bitter gourd are consumed together at an immature stage and contain anti-diabetic^5^ and hypoglycaemic compounds^6^, anti-carcinogenic and hypercholesterolemic^7,8^ and anti-HIV activity^9^. Bitter gourd fruits and seeds contain compounds like charantin^10^, momorcharin^11^ and momordicoside A and B^12^. The fruits of bitter gourd also possess antimicrobial^13^, antifertility^14^, antiviral^15^, and antiulcerogenic^16^, steroids^17^, anti-tumour^18^ properties and seeds of bitter gourd contain pyrimidine nucleoside vicine^19,20^ and nutritionally bitter gourd rich in ascorbic acid and iron among cucurbitaceous vegetable crops^21^.

Bitter gourd is grown in the tropical countries and preferences of the fruits vary from region to region for fruit colour, length, diameter, shape, size, tubercles, etc. Since immature fruits are sliced during the preparation of various Asian meals, exceptional internal fruit quality and uniform green peel colour are desirable. Fruit colour governs its marketability; green fruited types are in high demand in southern China, while white-fruited types are preferred in central China, similarly dark green to glossy green fruits are favoured in northern India, white fruits are preferred in southern India and eastern parts of India preferred small and dark green fruited types^22^. Long fruited types are preferred in north India, while medium-long fruited types are preferred in south India, whereas short fruited types are in high demand in eastern states of India^22^. Fruit length has a significant contribution to yield of any crop, so also in bitter gourd. However, the preference based on fruit length is a consumer’s choice. Further, many Asiatic countries including India directly cultivate the wild progenitors (traditionally small fruited *muricata* types) for consumption^23^. It was reported that top 0.1% SNPs associated with fruit size are not highly diverged between cultivar groups (long fruited types) and wild types (small fruited types) and therefore, the process of selection is slower in bitter gourd with introgressions between wild and cultivar groups preventing the strong and rapid fixation of domestication genes^23^.

Traditional phenotyping for high and consistent yield requires the evaluation for yield in multiple environments over several seasons, which is laborious, very expensive and time consuming^24^. The marker-assisted selection (MAS) greatly accelerates the breeding cycle and is a powerful molecular tool for selecting fruit traits and ultimately the yield. Two reports are available on the genetic linkage map for fruit traits in a bitter gourd by using the F_2_ mapping population^25^ and by using F_2:3_ mapping population^26^. The molecular basis of horticulturally important economic traits remains unexplored to date and no high-density genetic linkage map has been reported in bitter gourd. The scarcity of polymorphic molecular markers and the non-availability of whole-genome sequence information in the public domain till now have deprived development of the genetic linkage map and the application of molecular breeding in bitter gourd.

The precision of genetic map construction depends on the mapping population; recombinant inbred lines (RILs), near-isogenic lines (NILs), doubled haploid (DH) lines and backcross lines are highly efficient but are more laborious and time-consuming. In contrary, F_2_ population is the simplest and easiest to develop which is primarily based on Mendelian laws and therefore it was widely used for early genetic mapping and QTL analysis^27–29^, especially in non-model species with limited genetic research^30^. The genetic diversity analysis was reported earlier by many workers using various multi-locus dominant DNA markers such as RAPD^31,32^, ISSR^33^, and AFLP^34^ in bitter gourd. The microsatellites i.e.SSR markers are mostly preferred because of their co-dominance, repeatability and easy transferability even though the initial cost of development of these markers is very high^35,36^. However, the number of microsatellite markers available in *Momordica* species is few^37–42^. It is established that a greater number of markers are necessary for the development of a genetic map and marker-assisted selection^43^. Several reduced representation genome sequencing (RRGS) technologies were developed, such as restriction-sites associated DNA sequencing (RAD-seq)^44^, genotyping-by-sequencing (GBS)^45^, double digest restriction-sites associated DNA sequencing (ddRAD)^46^ and specific-locus amplified fragment sequencing (SLAF)^47^.

The genotyping-by-sequencing operates through restriction enzyme (RE) digestion; only a low percentage of the genome is sequenced but the fragments are normally well distributed across the genome^45^. It is applied recently as a very reliable tool for marker-assisted selection in accelerating crop improvement program^48^. It has been demonstrated efficiently for high-density map construction in several cucurbitaceous vegetable crops^49^. However, there was no high-density genetic map reported in a bitter gourd for yield and economic traits. Hence, the present study was conducted to generate a high-density QTL map using SNPs through GBS technology.

## Results

### Identification of SNPs and construction of linkage map

Sequencing all 93 libraries using each sample from 90 F_2_ individuals, one F_1_ and two parents yielded 93,926 SNP sites comprised of 18.4 GB data. About 40% of the variants were filtered out by imposing a missing value threshold and with 20% due to the other criteria imposed (at least one homozygous variant for a marker, global quality > 100, and only bi-allelic variants). Resulting SNPs were additionally filtered discarding those with heterozygosity. Finally, 2013 high-quality SNPs forming groups of SNPs with the same genotype for all samples were used for linkage map construction.

**Figure 1:**
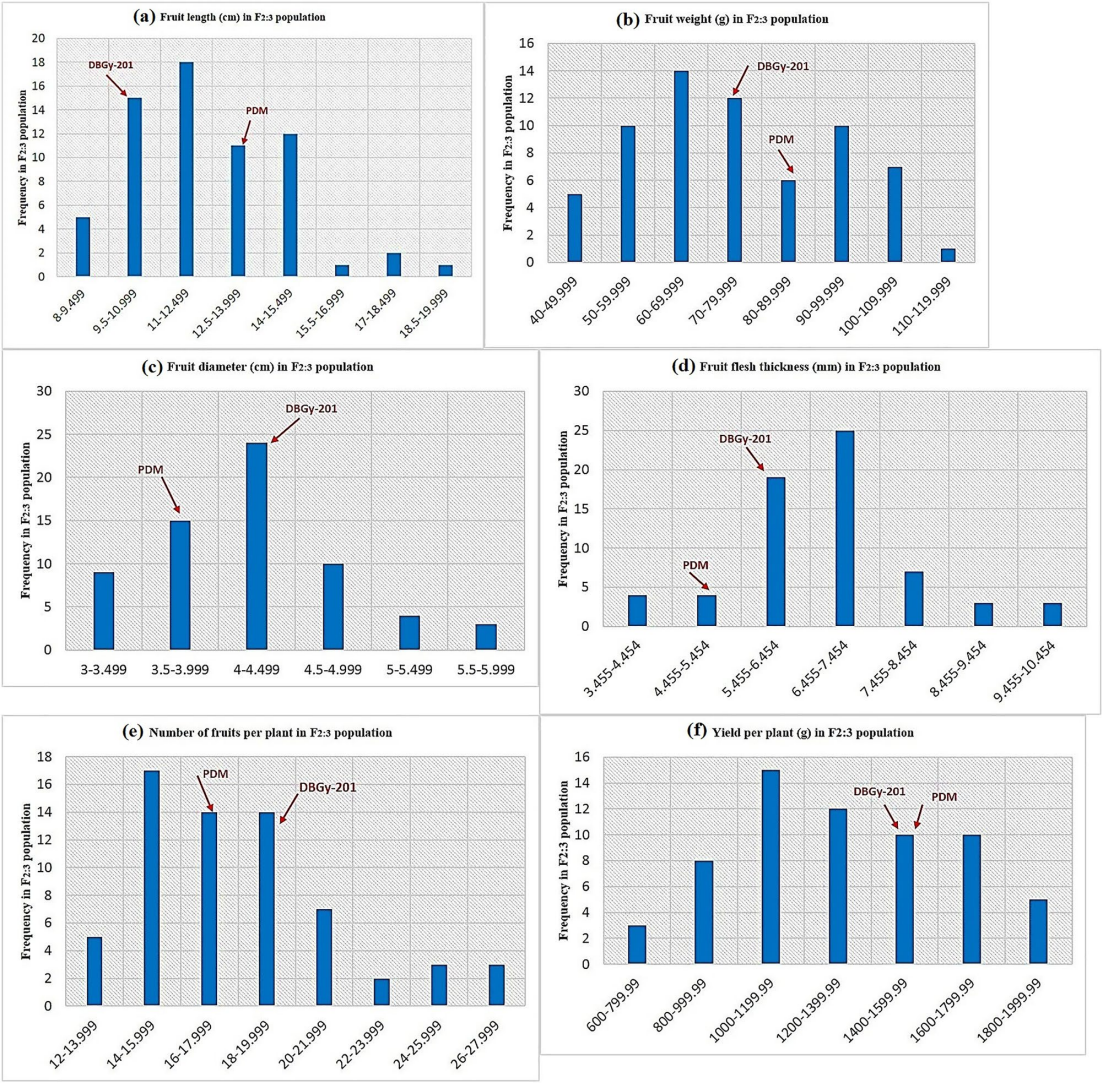
Frequency distribution pattern of mean trait values of F_2:3_ family (≈ 20 plants from each family by taking an average of five fruits per plant) from the cross DBGy-201 × Pusa Do Mausami.

The genetic map was constructed with 2013 high quality SNPs distributed across 20 linkage groups (Table 1, Fig. 1). The number of markers in individual LG ranged between 23 and 146 markers, with a mean of 100.6 markers per LG. The length of LG of ranged from 185.2 CM (LG 12) to 46.2 CM (LG-17) with a total genetic length of 2329.2 cM. Average genetic distance between successive markers was 1.16 CM, and the maximum spacing was estimated between the markers in LG-20 (2.92) and minimum in LG-4 (0.70).

**Table 1:** Summary of high-density SNP marker distribution on linkage groups in bitter gourd cross DBGy-201 × Pusa Do Mausami.

Linkage group (LG)	Length (cM)	No. of SNP markers	Average distance between markers (cM)	No. of QTLs
LG 1	138.3	146	0.95	4
LG 2	114.3	131	0.87	–
LG 3	94.3	131	0.72	1
LG 4	86.9	125	0.70	–
LG 5	155.7	123	1.27	2
LG 6	156.9	121	1.30	–
LG 7	134.1	119	1.13	–
LG 8	89.1	116	0.77	–
LG 9	158.9	116	1.37	–
LG 10	129.8	114	1.14	–
LG 11	169.0	108	1.57	–
LG 12	185.2	106	1.75	–
LG 13	119.7	105	1.14	1
LG 14	124.8	95	1.31	3
LG 15	135.5	85	1.59	3
LG 16	87.1	84	1.04	1
LG 17	46.2	59	0.78	–
LG 18	68.1	59	1.15	–
LG 19	68.2	47	1.45	–
LG 20	67.1	23	2.92	4
Total	2329.2	2013	–	19
Average	116.46	100.65	1.16	0.95

### Frequency distribution of major yield traits in F_2:3_ population

The phenotyping of six yield related traits was performed in F_2:3_ population derived from a cross DBGy-201 × Pusa Do Mausami. The variation for fruit traits in F_2:3_ population presented in Supplementary Fig. S1. Descriptive statistics (Supplementary Table S1) (range, mean, variance, standard deviation, skewness, and kurtosis), the broad sense heritability (h^2^b) estimates ranged from 0.70 (Fruit length) to 0.95 (Fruit weight). The mean performances and heritability (h^2^b) for fruit traits shown in Supplementary Table S1 and co-relation for six quantitative traits shown in Supplementary Table S2. Fruit length, fruit diameter, number of fruits per plant, fruit flesh thickness and fruit weight are major yield components. The frequency distribution pattern of fruit length in F_2:3_ population is shown in Fig. 1a. The frequency distribution showed a normal distribution pattern. The range of fruit length in F_2:3_ population was 8.14 cm to 18.59 cm with a mean value of 12.29 cm. short fruit length was more predominant in F_2:3_ population. Transgressive segregation was observed for higher fruit length with a total of 49.23% of transgressive segregants. The frequency distribution pattern of fruit diameter in F_2:3_ population is shown in Fig. 1b. The frequency distribution showed a normal distribution pattern. The range of fruit diameter in F_2:3_ population was 3.11 cm to 5.93 cm with the mean value of 4.18 cm with less fruit diameter was more predominant. Transgressive segregation was observed for higher fruit diameter with a total of 73.85% of transgressive segregants. The frequency distribution pattern of fruit weight in F_2:3_ population is shown in Fig. 1c. The frequency distribution showed a normal distribution pattern. The range of fruit weight in F_2:3_ population was 40.25 g to 116.39 g with the mean value of 74.95 g and the lower fruit weight was more predominant with a total of 86.15% of transgressive segregants. The frequency distribution pattern of fruit flesh thickness in F_2:3_ population is shown in Fig. 1d. The frequency distribution showed a normal distribution pattern. The range of fruit flesh thickness in F_2:3_ population was 3.46 mm to 10.20 mm with the mean value of 6.73 mm and thick fruit flesh was more predominant with a total of 73.85% of transgressive segregants.

The frequency distribution pattern of number of fruits per plant in F_2:3_ population is shown in Fig. 1e. The frequency distribution showed a normal distribution pattern. The range of number of fruits per plant in F_2:3_ population was 12.94 to 27.43 with the mean value of 18.10 and with a total of 93.85% of transgressive segregants. The frequency distribution pattern of yield per plant in F_2:3_ population is shown in Fig. 1f. The frequency distribution showed a normal distribution pattern. The range of yield per plant in F_2:3_ population was 665.37 g to 2446.09 g with a mean value of 1339 g and with a total of 86.15% of transgressive segregants. All the major yield components and yield in the F_2:3_ population were under continuous variation and followed a normal distribution, indicated that all the traits were controlled by polygenes.

### QTL detection for mapping yield traits

**Figure 2:**
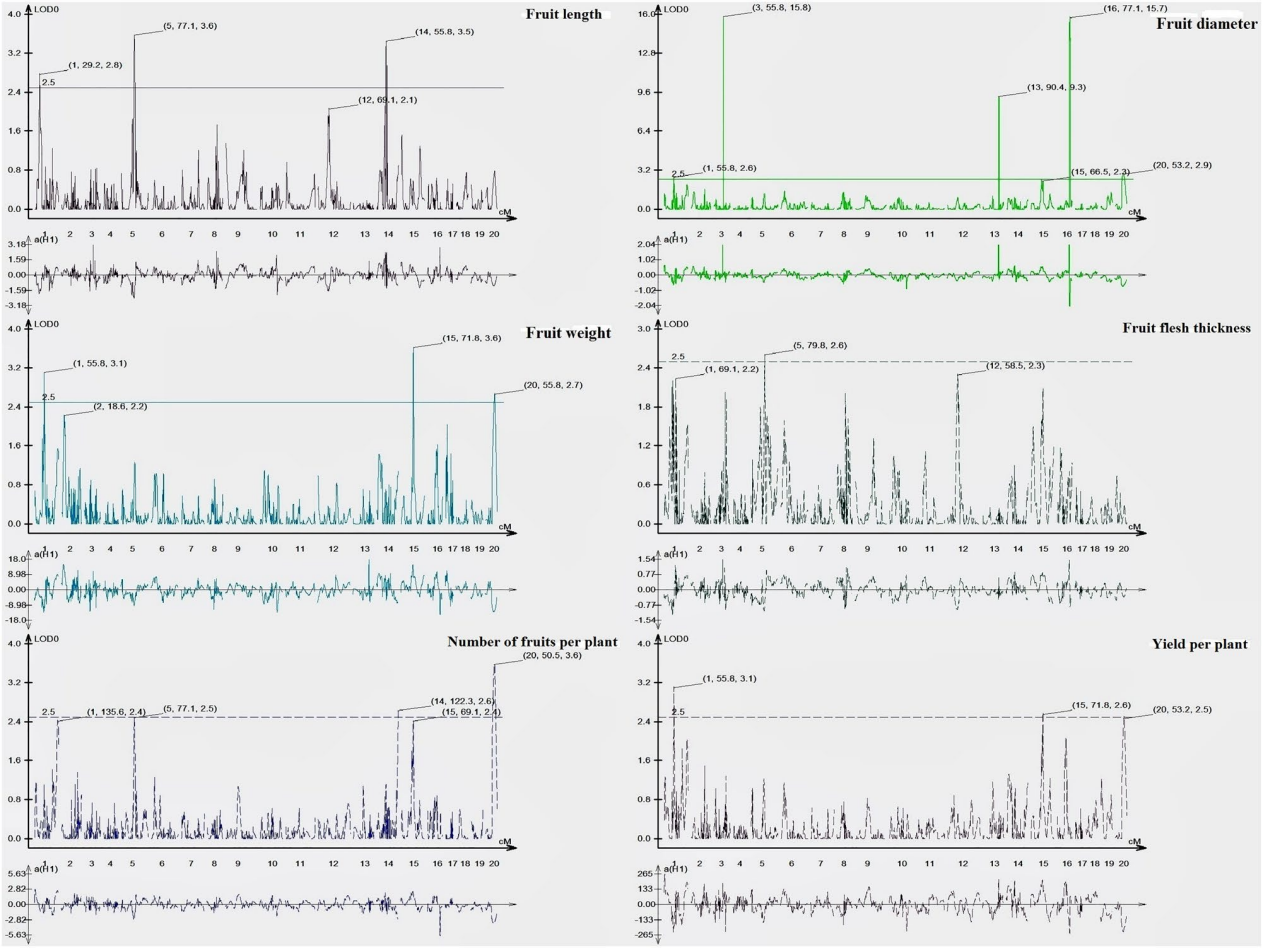
QTL LOD plot generated using QTL cartographer for the fruit traits of bitter gourd F_2:3_ family of a cross DBGy-201 × Pusa Do Mausami representing the linkage group, cM position and LOD value on the respective peaks.

Most of the yield traits were mapped across eight linkage groups out of 20 LG (Supplementary Fig. S2). The information about all these QTLs (explained variance, LOD peaks, flanking markers, and additive effects) is shown in Table 2 and QTL cartographer presented in Fig. 2. A total of 19 QTLs (9 QTLs with LOD > 3) were identified using composite interval mapping (CIM) based on the phenotyping of F_2:3_ families. The phenotypic variation (R^2^%) explained by these QTLs ranged from 0.09 to 32.65% and 6 major QTLs (R^2^ > 10%) were identified. Most of the QTLs identified in the present study were in adjacent regions in chromosomes LG-20, this may be due to a high correlation among the traits.

**Table 2:** QTL analysis of fruit traits in bitter gourd in F_2:3_ family lines.

S. No	Trait	QTL*	LG^y^	QTL Position (cM)	Flanking markers		Marker closest to peak	Closeness of marker to trait (cM)	R^2^ (%)	LOD	Additive effect^#^
					Left marker	Right marker					
1	Fruit length (cm)	*qFL1*	1	28.81	TP_3003	TP_2693	TP_3003	1.51	9.69	2.7	− 1.84
		*qFL5*	5	77.48	TP_11213	TP_11334	TP_11334	1.82	13.04	3.6	− 2.12
		*qFL14*	14	55.54	TP_67839	TP_68143	TP_67839	0.04	11.21	3.4	2.02
2	Fruit diameter (cm)	*qFD1*	1	55.8	TP_1877	TP_1459	TP_1459	0.10	9.63	2.6	− 0.60
		*qFD3*	3	55.86	TP_8845	TP_8817	TP_8817	0.74	0.10	15.8	0.06
		*qFD13*	13	90.49	TP_57231	TP_56896	TP_57231	0.49	0.27	9.3	0.10
		*qFD15*	15	66.54	TP_66813	TP_66819	TP_66813	0.04	6.16	2.5	0.49
		*qFD16*	16	77.12	TP_74581	TP_74591	TP_74591	4.78	32.65	15.7	− 1.12
		*qFD20*	20	53	TP_78027	TP_75976	TP_78027	0.50	9.46	2.8	− 0.61
3	Fruit weight (g)	*qFW1*	1	55.8	TP_1877	TP_1459	TP_1459	0.10	10.81	3.1	− 12.48
		*qFW15*	15	71.88	TP_67045	TP_67087	TP_67045	2.48	9.17	3.6	11.82
		*qFW20*	20	55.5	TP_78027	TP_75976	TP_78027	3.00	9.36	2.6	− 11.62
4	Fruit flesh thickness (mm)	*qFT14*	14	80.12	TP_68612	TP_68958	TP_68958	0.48	0.09	2.6	0.10
5	Number of fruits per plant	*qFN5*	5	76.98	TP_11213	TP_11334	TP_11334	2.32	9.07	2.5	− 2.66
		*qFN14*	14	122.25	TP_69841	TP_69846	TP_69841	1.85	8.20	2.6	− 2.51
		*qFN20*	20	50.47	TP_78598	TP_78027	TP_78027	2.03	13.81	3.6	− 3.27
6	Yield per plant (g)	*qYD1*	1	55.8	TP_1877	TP_1459	TP_1459	0.10	10.19	3.1	− 223.07
		*qYD15*	15	71.88	TP_67045	TP_67087	TP_67045	2.48	4.88	2.6	161.47
		*qYD20*	20	53	TP_78027	TP_75976	TP_78027	0.50	8.21	2.5	− 200.97

*QTL named as *qxxxy*, with “*xxx*” being the trait abbreviation, ‘*y*’ the number of the linkage group. # Additive effect was positive when the “DBGy-201” allele increased the trait score and was negative when the “Pusa Do Mausami” allele increased trait score. The 95% confidence interval of the QTL locations was determined with 2-LOD support interval which was defined by left and right markers.

### Fruit length (cm)

Two major and one minor QTLs were identified for fruit length, one major QTL on linkage group LG-5, one major QTL on the linkage group LG-14 and one minor QTLs on linkage group LG-1. The major QTL *qFL5* located between flanking markers TP_11213 and TP_11334 on LG-5 had shown LOD values of 3.60 and explained 13.04% of phenotyping variation (R^2^%). The major QTL *qFL14* was located between flanking markers TP_67839 and TP_68143 on LG-14, shown a LOD value of 3.40 and explained 11.21% of phenotyping variation (R^2^%). Two major QTLs together explained 24.25% of phenotyping variation for fruit length. The minor QTLs *qFL1* was located between flanking markers TP_3003 and TP_2693 on LG-1 had shown LOD values of 2.70 and explained 9.69% of phenotyping variation (R^2^%). Together, two major and one minor QTLs explained 33.94% of phenotyping variation for fruit length. The QTL *qFL14* showed a positive additive effect indicating allele for increasing fruit length which might have contributed by the female parent (DBGy-201) and the QTLs *qFL5* and, *qFL1* showed a negative additive effect indicating allele for increasing fruit length contributed by the male parent (Pusa Do Mausami).

### Fruit diameter (cm)

One major and five minor QTLs were identified for fruit diameter (cm), one major QTL on the linkage group LG-16, each one minor QTL on the linkage groups LG-1, LG-3, LG-13, LG-15 and LG-20. The major QTL *qFD16* was located between flanking markers TP_74581 and TP_74591 on LG-16 had shown LOD value of 15.70, explaining 32.65% of phenotyping variation (R^2^%). The minor QTL *qFD1* was located between flanking markers TP_1877 and TP_1459 on LG-1, had shown LOD value of 2.60, explaining 9.63% of phenotyping variation (R^2^%). The minor QTL *qFD20* located between flanking markers TP_78027 and TP_75976 on LG-20 had shown LOD values of 2.80 and explained 9.46% of phenotyping variation (R^2^%). Together with one major and five minor QTLs explained 58.27% of phenotyping variation for fruit diameter. One major and two minor QTLs showed a negative additive effect indicating allele for increasing fruit diameter contributed by the male parent (Pusa Do Mausami).

### Fruit weight (g)

One major and two minor QTLs were identified for fruit weight, one major QTL on the linkage group LG-1, one minor QTL on the linkage group LG-15 and one minor QTL on the linkage group LG-20. The major QTL *qFW1* was located between flanking markers TP_1877 and TP_1459 on LG-1 had shown LOD value of 3.10, explaining 10.81% of phenotyping variation (R^2^%). The minor QTL *qFW15* was located between flanking markers TP_67045 and TP_67087 on LG-15 had shown LOD value of 3.60, explaining 9.17% of phenotyping variation (R^2^%). The minor QTL *qFW20* was located between flanking markers TP_78027 and TP_75976 on LG-20, shown LOD value of 2.60 and explained 9.36% of phenotyping variation (R^2^%). Together with one major and two minor QTLs explained 29.34% of phenotyping variation for fruit weight. One major QTL *qFW1* and one minor QTL *qFW20* showed a negative additive effect indicating allele for increasing fruit weight contributed by the male parent (Pusa Do Mausami), whereas, one minor QTL *qFW15* showed a positive additive effect indicating allele for increasing fruit weight contributed by the female parent (DBGy-201).

### Fruit flesh thickness (mm)

One minor additive QTL were identified for fruit flesh thickness, on the linkage group LG-14. The minor QTL *qFT14* located between flanking markers TP_68612 and TP_68958 on LG-14, showed LOD value of 2.60, the minor QTL explained only 0.09% of phenotyping variation for fruit flesh thickness and showed positive additive effect indicating allele for increasing fruit flesh thickness contributed by the female parent (DBGy-201).

### Number of fruits per plant

One major and two minor QTLs were identified for number of fruits per plant, one major QTL on the linkage group LG-20, one minor QTL on the linkage group LG-5 and one minor QTL on the linkage group LG-14. The major QTL *qFN20* was located between flanking markers TP_78598 and TP_78027 on LG 20 showed LOD value of 3.60 and explaining 13.81% of phenotyping variation (R^2^%). The minor QTL *qFN5* were located between flanking markers TP_11213 and TP_11334 on LG-5 showed LOD values of 2.50 and explained 9.07% of phenotyping variation (R^2^%). The minor QTL *qFN14* was located between flanking markers TP_69841 and TP_69846 on LG-14 showed LOD value of 2.60 and explaining 8.20% of phenotyping variation (R^2^%). Together with one major and two minor QTLs explained 31.08% of phenotyping variation for a number of fruits per plant. One major QTL *qFN20* and two minor QTLs *qFN5* and *qFN14* showed negative additive effects indicated, alleles for an increasing number of fruits per plant contributed by the male parent (Pusa Do Mausami).

### Yield per plant (g)

One major and two minor QTLs were identified for yield per plant, one major QTL on the linkage group LG-1, two minor QTLs on the linkage group LG-15 and LG-20. The major QTL *qYD1* was located between flanking markers TP_1877 and TP_1459 on LG-1 had shown LOD value of 3.10 and explaining 10.19% of phenotyping variation (R^2^%). The minor QTL *qYD*15 located between the flanking markers TP_67045 and TP_67087 on LG-15 showed LOD value of 2.6 and explained 4.88% of phenotyping variation (R^2^%). The minor QTL *qYD20* located between flanking markers TP_78027 and TP_75976 on LG-20 had shown LOD value of 2.50 and explained 8.21% of phenotyping variation (R^2^%). Together with one major and two minor QTLs explained 23.28% of phenotyping variation for yield per plant. One major QTL *qYD1* and one minor QTLs *qYD20* were showed negative additive effects, indicated alleles for increasing yield per plant contributed by the male parent (Pusa Do Mausami).

## Discussion

At present, there is no precise report on QTL mapping for yield and yield related traits in bitter gourd and only a few studies have been reported using AFLP markers and mapped five of each qualitative and five quantitative trait loci^25,26^ constructed the genetic linkage map for 13 horticultural traits in bitter gourd. This lacuna of mapping of economic traits is the main hurdle for the utility of MAS in bitter gourd, so generating the high-density genetic mapping for yield components is need of the hour.

High throughput GBS technology with type-II restriction endonuclease *Ape*KI (GCWGC)^45^ was employed in this study to identify SNPs in F_2_ and F_2:3_ segregated populations for constructing the genetic map and QTL analysis for fruit yield and its attributing traits in bitter gourd. The whole-genome sequence of bitter gourd data was not available in the public domain, we performed non-reference based GBS with the UNEAK pipeline^50^. A total of 2013 SNP markers used to construct 20 linkage groups spanned over 2329.2 cM. A high-density genetic map was constructed using GBS technology in the present study which provided 0.86 marker/cM than previous reports, which was 0.30 mean marker density^51^, 0.42^52^ and 0.46^53^. In the present study, the genetic map had an excess of linkage groups (20) than the haploid chromosome number (n = 11)^54^ even though a significant number of markers (2013 SNPs) were binned to the genetic map. This may be due to the relatively small population size^55^, type of mapping population (F_2:3_ instead of RILs) and further, there is a need to enrich with more markers.

Inheritance of all fruit traits and yield under study had shown continuous variation and it indicates the traits were polygenic. There was a high correlation between fruit yield and fruit weight (0.706) and also between fruit diameter and fruit weight (0.702). The correlation ranged from − 0.313 to 0.706, which was at a significant level. The six quantitative traits were mapped and 19 QTLs were identified using composite interval mapping (CIM) with an average of 3 QTLs per trait. Out of 19 QTLs, 12 QTLs were derived from ‘Pusa Do Mausami’ showed a negative additive effect and seven QTLs were derived from ‘DBGy-201’ showed a positive additive effect.

Yield components like fruit length, fruit diameter, fruit weight and number of fruits per plant greatly contribute to total fruit yield per plant^56,57^. Two major QTLs together explained 24.25% of phenotyping variation for fruit length. The QTL *qFL14* had shown positive additive effects, thus DBGy-201 alleles from this QTL may have increased the fruit length by 2.02 cm, whereas QTLs *qFL5* showed negative additive effects, thus Pusa Do Mausami alleles from these QTLs increased the fruit length by 2.21 cm and 2.12 cm respectively. There were also some other QTL loci screened for fruit length in bitter gourd as two QTLs^25^ and four QTLs^26^. Three QTLs for immature fruit length and four QTLs for mature fruit length have been detected in zucchini^58^ and one QTL in melon^58^ based on GBS technology.

One major QTL, *qFD16* was identified for fruit diameter, explained 32.65% of phenotyping variation, and showed a negative additive effect, thus Pusa Do Mausami alleles from this QTL increased the fruit diameter by 1.12 cm. Kole et al.,^25^ and Wang and Xiang^26^ had identified one and five QTLs, respectively for fruit diameter in bitter gourd^25,26^. One QTL for immature fruit width and two QTLs for mature fruit width were identified in zucchini based on GBS technology^58^. For fruit weight one major QTL, *qFW1* identified and explained 10.81% of phenotyping variation and showed a negative additive effect, thus Pusa Do Mausami alleles from this QTL increased the fruit weight by 12.48 g. In bitter gourd, one QTL^25^ and four QTLs^26^ reported for fruit weight.

One major and two minor QTLs together explained 31.08% of phenotyping variation for number of fruits per plant. One major QTL *qFN20* and two minor QTLs *qFN5* and *qFN14* shown negative additive effects, thus Pusa Do Mausami alleles from these QTLs increased the number of fruits per plant by 3.27, 2.66, 2.56 and 2.51 respectively. Similarly, four QTLs^25^ and three QTLs^26^ mapped in bitter gourd and seven QTLs in cucumber^60^ for number of fruits per plant.

Fruit flesh thickness is an important trait for bitter gourd fruit quality and a central determinant of yield; that is, the thicker the fruit flesh, the greater the edible portion of the bitter gourd fruit. One minor QTL explained only 0.09% of phenotyping variation for fruit flesh thickness. The minor QTL *qFT14* shown positive additive effects, thus DBGy-201 alleles from these QTLs increased the fruit flesh thickness by 0.10 mm each. Two QTLs^26^ mapped in bitter gourd for fruit flesh thickness. Xuewen and his associates^61^ did the mapping in cucumber for fruit flesh thickness on chromosome 2 (QTL fft2.1) of the 0.19 Mb long region. One QTL was identified for fruit flesh thickness in melon^59^ based on GBS technology.

Yield per plant is itself not a trait; it is a product of complex interaction of many fruit traits such as fruit length, fruit diameter, fruit weight, fruit number and fruit flesh thickness along with environmental interactions. Both the parents, DBGy-201 and Pusa Do Mausami have fallen in the same class of performance for yield per plant, but F_2:3_ population has shown high transgressive segregants due to wider differences for sex form and plant architecture and fruit-related traits. One major and two minor QTLs together explained 23.28% of phenotyping variation for yield per plant. One major QTL *qYD1* and the minor QTLs *qYD20* shown negative additive effect, thus Pusa Do Mausami alleles from this QTL increased the yield per plant by 223.07 g, 200.97 g and 199.50 g respectively. Likewise, four QTLs^25^ and two QTLs^26^ mapped in a bitter gourd for yield per plant.

## Conclusion

QTL analysis was performed for six major yield contributing traits in bitter gourd using F_2:3_ mapping population derived from the cross DBGy-201 × Pusa Do Mausami. Two major QTLs together explained 24.25% of phenotyping variation for fruit length whereas one major QTL, *qFD16* was identified for fruit diameter, explained 32.65% of phenotyping variation. Similarly, one major QTL *qFW1* explained 10.81% of phenotyping variation for fruit weight and 1 major QTL *qFN20* with two minor QTLs *qFN5* and *qFN14* together explained 31.08% of phenotyping variation for number of fruits per plant. One major QTL *qYD1* and two minor QTLs *qYD15* and *qYD20* explained 23.28% of phenotyping variation for yield per plant. The QTLs identified in the present study will be helpful in marker-assisted selection and molecular breeding in bitter gourd crop improvement.

## Materials and methods

### ***Development of F***_***2:3***_*** mapping population***

A gynoecious line DBGy-201 (PVGy-201) was crossed with monoecious cultivar Pusa Do Mausami (PDM) of bitter gourd and 65 F_2:3_ mapping population was developed. The parents were crossed to develop F_1_ seeds and the F_1_ plants selfed to develop F_2_ population (90) at the vegetable research farm of IARI, New Delhi, India during spring–summer (February- May). Further, the F_2_ population was selfed individually to develop 65 F_2:3_ families (due to difficulty in getting selfed seeds from some plants, all F_2_ population unable to produce F_2:3_ families). Along with two parents, 65 F_2:3_ families were planted during spring–summer (February- May) to study the fruit yield and its attributing traits. About 20 F_2:3_ seeds from each family were sown in a single row with three replications, following recommended agronomic practices. The phenotyping data of parental lines, F_1_, and F_2:3_ families were collected on an individual basis, 20 plants in each parent, 30 plants in F_1_ and 65 families in F_2:3_ population (≈ 20 plants from each family) by taking an average of five fruits per plant.

### Genomic DNA extraction and quantification

Genomic DNA was extracted from young leaf tissues of both the parents, F_1_ and F_2_ populations following the modified CTAB method^62^. The quantity and quality of extracted genomic DNA was checked with help of a spectrophotometer (NanoDrop 8000; Thermo Fisher Scientific). An estimated concentration of 100 ng/ µL of total genomic DNA was used to prepare each library.

### Choosing the most suitable restriction enzyme (RE)

RE that leaves an overhang of more than one nucleotide is extremely useful for efficient adapter ligation to insert DNA^46^. Different REs like *Ape*KI, *Eco*T22I, *Msp*I and *Pst*I were screened to choose the most appropriate RE for bitter gourd GBS library preparation. Among these *Ape*kI enzyme gave the best library fragment distribution with uniform coverage and hence was chosen for library preparation for all bitter gourd samples (both the parents. F_1_ and 90 F_2_ plants). *Ape*KI is a type II restriction endonuclease (partially methylation sensitive) that recognizes a degenerate 5 bp sequence (GCWGC, where W is A or T) and creates a 5′ overhang (3 bp)^45^. *Ape*KI will not cut if the 3′ base of the recognition sequence on both strands is 5-methylcytosine. Ninety-six plex library preparation protocol was designed^46^ to conduct the present experiment.

### Adapters for GBS

Two types of adapters were used for GBS analysis, the “barcode” adapter terminates with 5 to 10 bp barcode on top strand at 3′ end. Barcode with 3 bp overhangs at the 5′ end on its bottom strand that is complementary to the “sticky” end generated by *Ape*KI (CWG)^49^. The oligonucleotide sequences with two barcode adapters are:

- 5′-ACACTCTTTCCCTACACGACGCTCTTCCGATCTxxxx.
- 5′-CWGyyyyAGATCGGAAGAGCGTCGTGTAGGGAAAGAGTGT.

where “xxxx” and “yyyy” denotes the barcode and its complement with sequences.

An *Ape*KI-compatible sticky end present only on the second, or “common”, adapter:

- 5′-CWGAGATCGGAAGAGCGGTTCAGCAGGAATGCCGAG
- 5′-CTCGGCATTCCTGCTGAACCGCTCTTCCGATCT

Adapters were designed based on the recognition site of *Ape*KI did not occur in any other adapter sequences and was not regenerated after ligation to genomic DNA. Single-end adapters were used for library preparation. For preparing each library we have used 94 samples (90 F_2,_ each one from two parents, F_1_ and one negative control) for tagging different barcodes, which have a variable length 5 to10 nucleotides.

### Illumina sequencing and raw sequence data processing

Ninety-three libraries (90 F_2_ and two parents and one F_1_) were sequenced using the protocol^49^; along with one negative control. The reads were filtered by following protocol^46^; perfectly matched to the one of the barcodes and the expected four-base remnant of the *Ape*KI cut site (CWGC), no adapter dimers and reads with no “NS” (minimum Q score of 10) across the first 72 bases^49^. The sequence reads from raw data FASTQ files have been processed via *de-novo* GBS analysis pipeline as implemented in UNEAK^49^. Software for sequence filtering and mapping analysis is a part of the TASSEL package and is available on SourceForge (http://sourceforge.net/projects/tassel/). The complete genomic data deposited at NCBI (the SRA number was SUB4509570 and the Bio project ID was PRJNA493717).

### Construction of genetic linkage map

The genotypic data matrix developed on the basis of the polymorphic SNP score pattern. The linkage map was constructed with a minimum and maximum LOD threshold and the χ^2^ test was performed using JOINMAP 4.1 by following the procedure^49^. The linkage groups were converted to a LOD map using a regression algorithm with the following settings: linkages with recombination frequency (< 0.49), LOD (> 0.01) threshold for removing loci for goodness-of-fit jumping (5.0) and performing a ripple after adding 2 loci. The distance was calculated by the Kosambi’s mapping function and the linkage groups were drawn using the Map Chart.

### QTL analysis

The QTL analysis was carried out on the set of 65 F_2:3_ families with phenotypic data for fruit yield and its attributing traits and the genotypic data consisted of marker loci. A minimum of 5 fruits per plant from 20 plants in each F_2:3_ family with total of 100 fruits from each family. The QTLs were detected with the WinQTL Cartographer v2.5^63^ software by composite interval mapping (CIM)^64,65^. The statistical significance thresholds were used to declare the presence of QTLs were determined by 1000 random permutations with a genome-wide type I error rate of 5% (*p* = 0.05)^66^. The 95% confidence interval of the QTL locations was determined with 2-LOD support interval which was defined by left and right markers (Table 2)^67^. The additive effect of the detected QTLs was also estimated by the WinQTL Cartographer v2.5. The R^2^ value from this analysis was accepted as the percent phenotypic variance explained by the locus.

## Supplementary Information

Supplementary Information 1.
