# Author Correction: Genetic analysis and QTL mapping of yield and fruit traits in bitter gourd (*Momordica charantia* L.)

**DOI:** 10.1038/s41598-021-91764-5

**Published:** 2021-06-10

**Authors:** P. Gangadhara Rao, T. K. Behera, Ambika B. Gaikwad, A. D. Munshi, Arpita Srivastava, G. Boopalakrishnan, Vinod

**Affiliations:** 1 Division of Vegetable Science, ICAR-Indian Agricultural Research Institute, New Delhi, 110012 India; 2 ICAR-National Bureau of Plant Genetic Resources, New Delhi, 110012 India; 3 Division of Genetics, ICAR-Indian Agricultural Research Institute, New Delhi, 110012 India

Correction to: *Scientific Reports* 10.1038/s41598-021-83548-8, published online 18 February 2021

This Article contains errors in Reference 4, which is incorrectly given as:

Behera, K. T. DBGy-201 and DBGy-202: two gynoecious lines in bitter gourd (*Momordica charantia* L.) isolated from indigenous source. *Indian J. Genet.* **66**, 61–62 (2006).

The correct reference is listed below:

Behera, T. K., Dey, S. S. & Sirohi, P. S. DBGy-201 and DBGy-202: two gynoecious lines in bitter gourd (*Momordica charantia* L.) isolated from indigenous source. *Indian J. Genet.* **66**, 61–62 (2006).

