# Supplementary material for: Genetic analysis and QTL mapping of yield and fruit traits in bitter gourd (Momordica charantia L.)
Source: Sci Rep. 2021 Feb 18;11:4109. doi: 10.1038/s41598-021-83548-8 (PMC7893057; doi:10.1038/s41598-021-83548-8)
Supplement: Supplementary file 1 — Supplementary Information 1. [file 41598_2021_83548_MOESM1_ESM.docx]

**Genetic analysis and QTL mapping of yield and fruit traits in bitter gourd (*Momordica charantia* L.)**

P. Gangadhara Rao^1^, T. K. Behera*^1^, Ambika B. Gaikwad^2^, A. D. Munshi^1^, Arpita Srivastava^1^, G. Boopalakrishnan^1^ and Vinod.^3^

^1^Division of Vegetable Science, ICAR-Indian Agricultural Research Institute, New Delhi 110012, India

^2^ICAR-National Bureau of Plant Genetic Resources, New Delhi 110012, India

^3^Division of Genetics, ICAR-Indian Agricultural Research Institute, New Delhi 110012, India

*Corresponding author: [tusar@rediffmail.com](mailto:tusar@rediffmail.com); tusariari@gmail.com


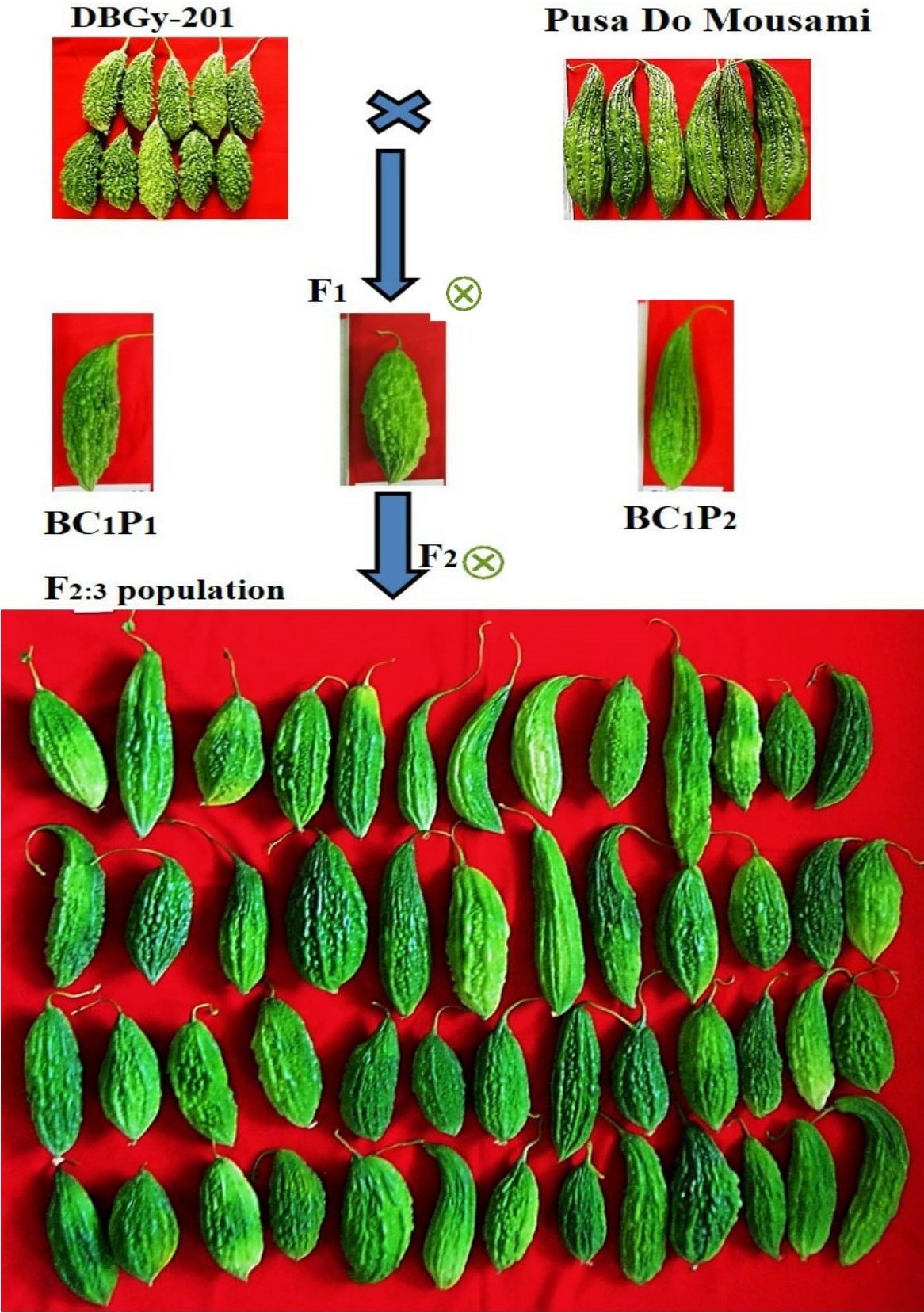


**Supplementary Fig. S1**. The variation for fruit traits of F_2:3_ family in cross DBGy-201 × Pusa Do Mausami.

**
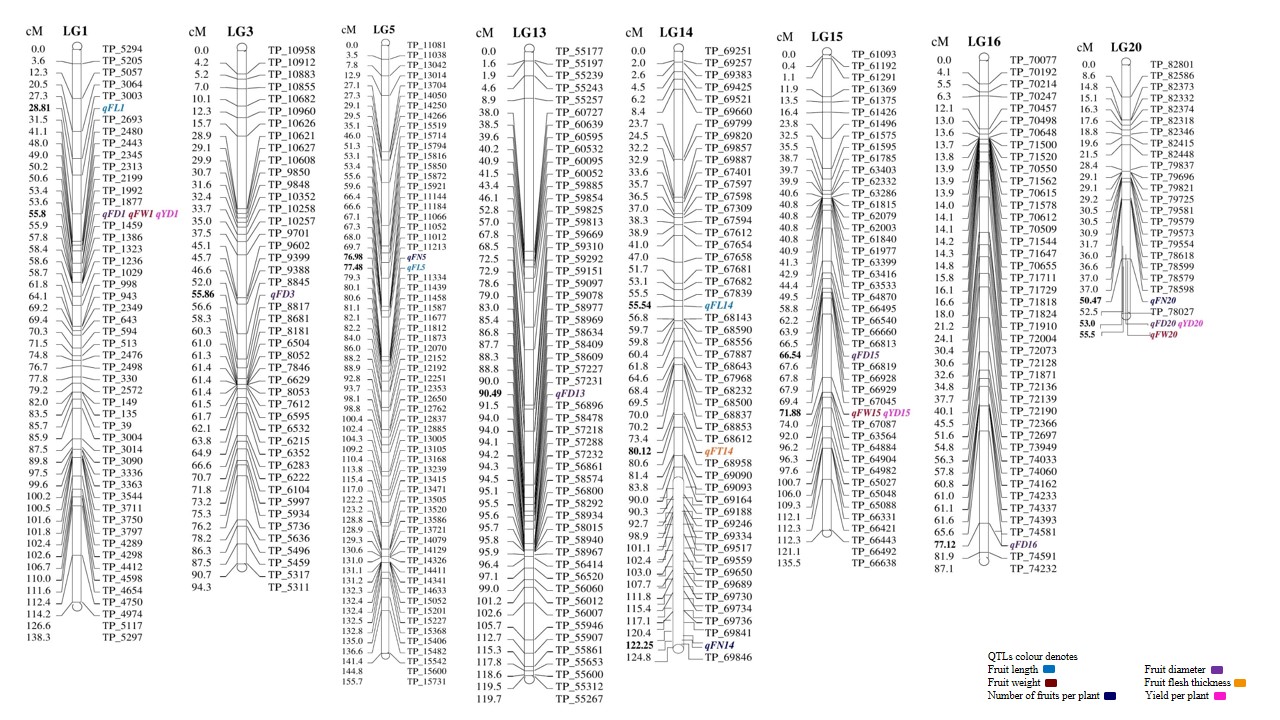
Supplementary Fig. S2**. Linkage map of bitter gourd of a cross (DBGy-201 × Pusa Do Mausami) using F_2:3_ family along with peak positions of quantitative trait loci (QTLs) for fruit traits.

**Supplementary Table S1**. Mean performances of Parents, F_1_ and descriptive statistics for fruit traits of F_2:3_ family of cross DBGy-201 × Pusa Do Mausami.

| S.  No. | Particulars | Parents | | F_1_ | F_2:3_ family | | | | | | | | |
| --- | --- | --- | --- | --- | --- | --- | --- | --- | --- | --- | --- | --- | --- |
|  | Trait | Female | Male |  | Range | Mean | LSD 0.05 | Variance | Skewness | Kurtosis | CV (%) | Transgressive segregants (%) | Heritability (h^2^b) |
| 1 | Fruit length (cm) | 9.62 | 12.67 | 12.31 | 8.14-18.59 | 12.29 | 2.23 | 5.14 | 0.65 | 3.14 | 18.14 | 49.23 | 0.70 |
| 2 | Fruit diameter (cm) | 4.17 | 3.90 | 4.15 | 3.11-5.93 | 4.18 | 0.64 | 0.41 | 0.65 | 3.11 | 15.31 | 73.85 | 0.80 |
| 3 | Fruit weight (g) | 74.86 | 87.16 | 93.17 | 40.25-116.39 | 74.95 | 18.79 | 353.00 | 0.31 | 2.10 | 25.07 | 86.15 | 0.95 |
| 4 | Fruit flesh thickness (mm) | 6.20 | 5.23 | 7.59 | 3.46-10.20 | 6.73 | 1.32 | 1.75 | 0.21 | 4.22 | 19.61 | 73.85 | 0.84 |
| 5 | Number of fruits per plant | 18.97 | 17.81 | 28.76 | 12.94-27.43 | 18.10 | 3.46 | 11.97 | 0.91 | 3.33 | 19.12 | 93.85 | 0.80 |
| 6 | Yield per plant (g) | 1419.29 | 1554.78 | 2679.44 | 665.37-2446.09 | 1339.00 | 364.50 | 132900.00 | 0.58 | 3.30 | 27.22 | 86.15 | 0.92 |

Mean performances of Parents and F1 for fruit traits. Mean, Range, LSD, Variance, Skewness, Kurtosis, CV%, Transgressive segregants %, and Heritability (h^2^b) of F_2:3_ family. LSD 0.05 = least significant difference at 5% confidence level, h^2^b = broad sense heritability.

**Supplementary Table S2**. Pearson’s correlation coefficient of fruit traits of F_2:3_ family of cross DBGy-201 × Pusa Do Mausami.

| Trait | Fruit length (cm) | Fruit diameter (cm) | Fruit weight (g) | Fruit flesh thickness (mm) | Number of fruits per plant | Yield per plant (g) |
| --- | --- | --- | --- | --- | --- | --- |
| Fruit length (cm) | 1.000 | 0.120 | 0.572** | 0.328** | -0.313** | 0.295** |
| Fruit diameter (cm) |  | 1.000 | 0.702** | 0.586** | -0.201* | 0.480** |
| Fruit weight (g) |  |  | 1.000 | 0.645** | -0.257** | 0.706** |
| Fruit flesh thickness (mm) |  |  |  | 1.000 | -0.290** | 0.373** |
| Number of fruits per plant |  |  |  |  | 1.000 | 0.480** |
| Yield per plant (g) |  |  |  |  |  | 1.000 |

∗∗Significant at 1% level
